# Humeral bone grafting in stemless shoulder arthroplasty

**DOI:** 10.1007/s11678-017-0424-2

**Published:** 2017-08-03

**Authors:** Fabian Plachel, Markus Scheibel

**Affiliations:** Department of Shoulder and Elbow Surgery, Center for Musculoskeletal Surgery, Charité – Universitätsmedizin Berlin, Augustenburger Platz 1, 13353 Berlin, Germany

## Background

**Fig. 1:**
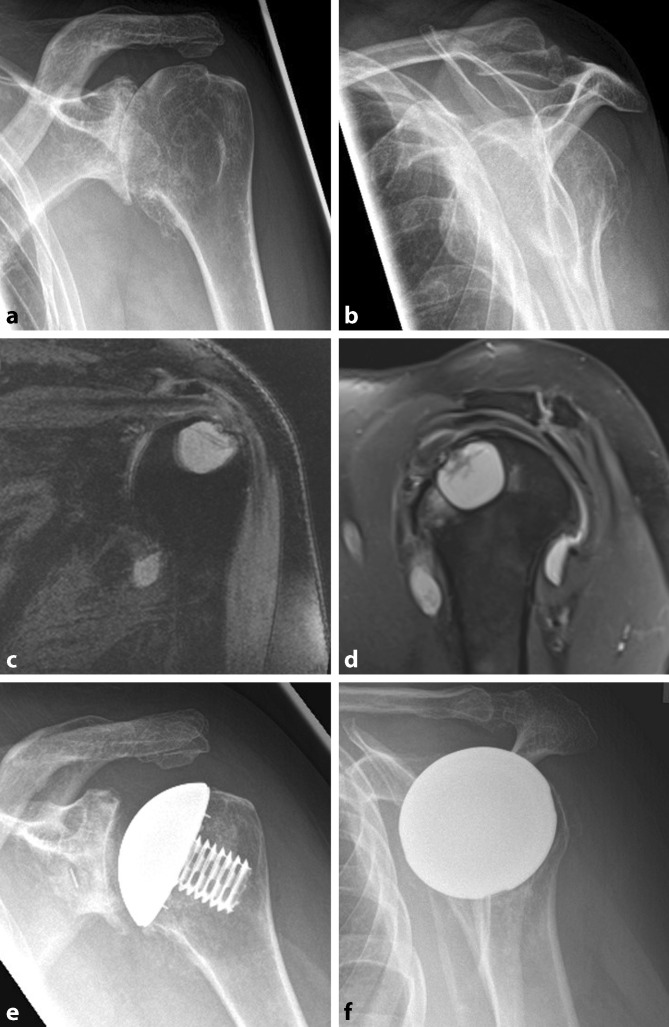
**a**, **b** Conventional radiographic imaging of a left shoulder revealed severe primary osteoarthritis with extensive humeral and glenoidal osteophyte formation. **c**, **d** Further magnetic resonance imaging of the affected shoulder showed a large bone cyst at the posterosuperior portion of the proximal humerus, yet the rotator cuff was figured to be intact. **e**, **f** Postoperative radiographic imaging after total shoulder arthroplasty with a stemless humeral component and humeral autograft

Stemless shoulder arthroplasty with metaphyseal fixation of the humeral component is increasingly used in the treatment of primary or secondary osteoarthritis, achieving significant pain relief and improving both range of motion and patient satisfaction [1, 4, 6]. The major advantages of the stemless humeral design are preservation of humeral bone stock, anatomical reconstruction regardless of humeral malalignment, fewer stem-related complications, and ease of revision [2]. Hawi et al. recently showed good long-term clinical and radiological outcomes with a revision rate of approximately 7% without humeral implant-related complications [6]. Currently, poor bone quality, including osteoporosis or metaphyseal cystic changes, is described to be a contraindication for stemless shoulder prostheses [5]. Thus, it is generally recommended to switch to a stemmed prosthesis to provide bone ingrowth and primary stability. The purpose of the following technique is to embrace the advantages of the stemless design when treating severe primary or secondary osteoarthritis, even in the presence of a large humeral bone defect, using the Eclipse prosthesis (Arthrex, Naples, FL, USA) combined with a humeral autograft (Fig. 1a–d).

## Technical note

### Step 1: Exposure of the glenohumeral joint

**Fig. 2a–o:**
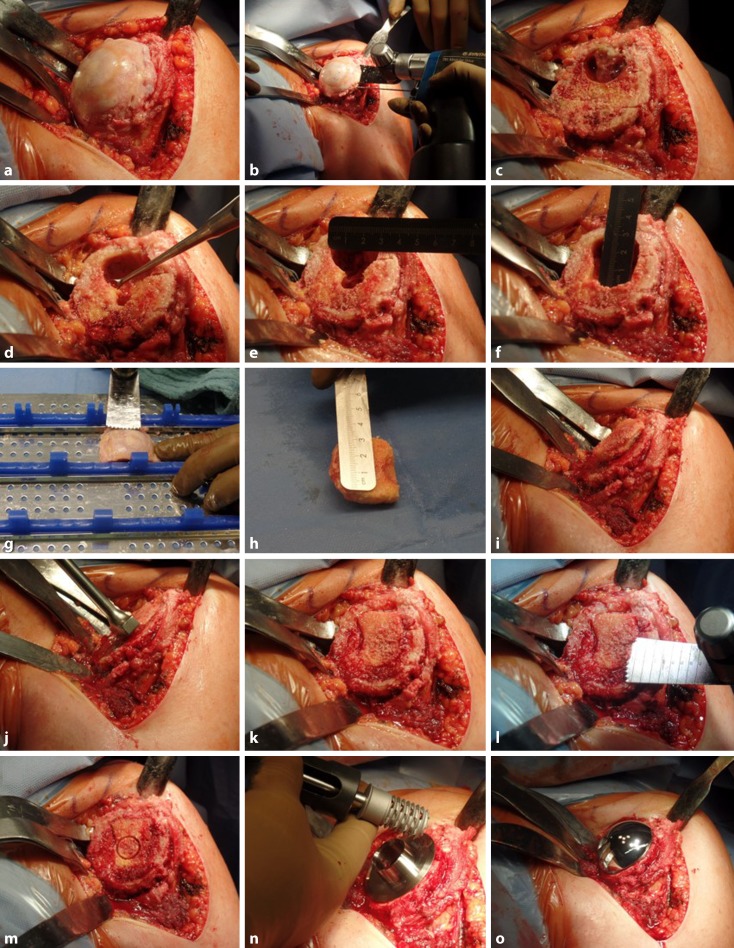
Step-by-step illustration of humeral bone grafting in stemless shoulder arthroplasty

The patient is placed in a beach chair position. The affected arm is draped free and placed on a sterile side table attached to the side of the operating table. A deltopectoral skin incision extending from the coracoid process to the lateral aspect of the deltoid insertion is made. After identifying the cephalic vein, the deltopectoral interval is prepared until the deltopectoral fascia is encountered. A detailed subdeltoid adhesiolysis and subacromial bursectomy are performed digitally, followed by vertical incision of the deltopectoral fascia lateral to the conjoint tendon to reveal the subscapularis muscle. By rotating the affected arm externally, both the tendon insertion and the musculotendinous unit of the subscapularis muscle are exposed. The subscapularis tendon is entirely released approximately 0.5–1 cm medial to its insertion on the lesser tuberosity parallel and medial to the bicipital groove. The detached tendon is secured by up to four nonabsorbable sutures using a modified Mason–Allen technique. A tenolysis is followed by release of the capsule to facilitate glenohumeral exposure and achieve better mobility. Finally, the affected arm is further externally rotated and adducted to gently dislocate the humeral head from the glenoid until the entire humeral articular surface is visible (Fig. 2a).

### Step 2: Humeral head osteotomy

The humeral osteophytes are removed completely to identify the anatomic neck of the humerus. Once the centromedullary humeral resection guide has been placed in a standard method, the humeral head is resected with an oscillating saw at the appropriate level (Fig. 2b). The cut surface is then palpated digitally to examine bone quality within the proximal humeral metaphysis (“thumb test” [3]).

### Step 3: Examination of the humeral bony defect

As shown in Fig. 2c, a large solitary bone cyst is detected within the lateral aspect of the metaphyseal cut surface. After the cyst has been thoroughly debrided, curettage of the fibrous membrane is performed using a sharp spoon to explore the exact location, extent, and configuration of the bony defect (Fig. 2d). The diameter and depth of the resultant humeral lesion are measured with a ruler (Fig. 2e, f).

### Step 4: Autograft preparation and impaction

A spongious autograft is harvested from the resected humeral head depending on the individual extent of the bony defect. It is important to gently remove the cortical bone with a saw or a burr (Fig. 2g, h). The shaped cancellous autograft is then impacted flush with the humeral cut surface (Fig. 2i–k). A saw is used to manually create a flat surface ensuring a circumferential contact area (Fig. 2l).

### Step 5: Preparation of the central screw hole

The drill template is matched with the cortical rim and then placed on the resected plane to prepare the central hole for the cage screw using a hand coring reamer. It is important to manually ream the bone due to the underlying autograft (Fig. 2m). The length of the screw is measured using the standard devices and technique. A resection protector is used while preparing the glenoid.

The glenoid is prepared using a standard technique. Once the glenoid component has been implanted, the selected trunion is placed on the humeral cut and fixed with the appropriate cage screw (Fig. 2n). While tightening the screw, the graft is pressed into the metaphysis via the trunion. The humeral head prosthesis is impacted and the procedure is finalized by repositioning the humeral head and reattachment of the subscapularis tendon using both transosseous and tendon-to-tendon sutures (Fig. 2o).

Postoperatively, the shoulder is immobilized in a sling for 6 weeks, and passive range-of-motion exercises for abduction, flexion, and internal rotation are begun 2 weeks after surgery. After 6 weeks, conventional radiographic imaging is performed to assess component position and to check for signs of prosthetic loosening (Fig. 1e, f).

## Discussion

The main concern regarding the metaphyseal fixation of the humeral stemless components is poor bone quality due to osteoporosis, osteopenia, or metabolic bone diseases. The second major problem is the presence of a bone defect in the proximal humerus such as a subchondral cyst formation, which may be found in many articular diseases [7]. In those cases, a stemmed prosthesis is currently a viable option to utilize diaphyseal fixation and enable primary implant stability, even though several stem-related complications have been reported, including intraoperative humeral fractures and postoperative stress-shielding, stem loosening, or osteolysis [8].

Among the various stemless humeral prostheses available to treat primary and secondary osteoarthritis, the Eclipse prosthesis is the only stemless system providing metaphyseal fixation by a fully threaded cylindrical central cage screw. Compared to the prostheses which are implanted using an impaction technique, the screw-in application is thought to be more bone preserving and leads to excellent compression of the autograft into the metaphysis.

Our technique represents a reliable option when treating patients with osteoarthritis in combination with a significant metaphyseal humeral bone defect using a stemless humeral prosthesis, together with a readily available humeral autograft.

## Caption Electronic Supplementary Material

Humeral bone grafting in stemless shoulder arthroplasty
